# Metronomic Temozolomide (mTMZ) and Bevacizumab—The Safe and Effective Frontier for Treating Metastatic Neuroendocrine Tumors (NETs): A Single-Center Experience

**DOI:** 10.3390/cancers15235688

**Published:** 2023-12-01

**Authors:** Çağlar Ünal, Sezer Sağlam

**Affiliations:** 1 Division of Medical Oncology, Department of Internal Medicine, Kartal Dr. Lütfi Kırdar City Hospital, İstanbul 34870, Turkey; 2 Division of Medical Oncology, Department of Internal Medicine, Demiroglu Bilim University, İstanbul 34870, Turkey; saglam@istanbul.edu.tr

**Keywords:** bevacizumab, neuroendocrine neoplasia, neuroendocrine tumors, metronomic temozolomide, mTMZ, CAPTEM

## Abstract

**Simple Summary:**

A recent study explored the use of metronomic temozolomide (mTMZ) and bevacizumab in treating metastatic neuroendocrine tumors (NETs). By analyzing data from 30 patients, this research provides insights into survival rates, tolerance to the treatment, and the spectrum of side effects observed. The results, showing promise, warrant further investigation into how this treatment combination compares with the established temozolomide–capecitabine (CAPTEM) regimen.

**Abstract:**

Addressing the persistent challenges in treating metastatic neuroendocrine tumors (NETs) demands ongoing refinement and innovation in therapeutic strategies. This study investigates the potential advantages of combining metronomic temozolomide (mTMZ) with bevacizumab for patients diagnosed with metastatic NETs, particularly focusing on those with a Ki-67 index under 55%. Data from 30 patients were analyzed, using key performance indicators such as progression-free survival (PFS), overall survival (OS), and response rates to therapy, to gauge the treatment’s efficacy. The results were encouraging: the median PFS recorded was 16.3 months, and the OS was 25.9 months. The disease control rate (DCR) reached an impressive 86.7%, and the objective response rate (ORR) stood at 63.3%. The treatment regimen was well-tolerated, with no reported instances of grade 4 toxicities. Such a safety profile indicates that this regimen may be particularly advantageous for older, fragile patients who might struggle with conventional dosage levels. These initial findings suggest that the mTMZ and bevacizumab combination could potentially rival the conventional temozolomide–capecitabine therapy in managing metastatic NETs. We aimed to meticulously assess the efficacy of the mTMZ and bevacizumab combination in treating metastatic NETs. Given the initial promising results, a more conclusive understanding of its efficacy will require further research through larger, multicenter prospective clinical trials.

## 1. Introduction

Neuroendocrine neoplasms (NENs) represent a diverse array of rare tumors, originating from neuroendocrine cells. These neoplasms can develop in a variety of organs throughout the body [1]. With advancements in diagnostic radiology and pathology, there has been a marked increase in the identification of these tumors [1]. The incidence and prevalence of NENs are reported to be 5.86 and 6.98 per 100,000 individuals, respectively, and these figures are continually escalating [2]. Intriguingly, a significant proportion of neuroendocrine tumors are asymptomatic and non-functioning. They are frequently discovered serendipitously during autopsies, or their diagnosis is discerned retrospectively following procedures such as appendicectomies or liver biopsies [3]. The digestive system has been identified as the predominant site for these neoplasms, followed closely by the lungs [4]. The classification of NENs generally hinges on the primary tumor’s location, its histological grade, and Ki-67 values [5]. Notably, while patients with locally advanced NENs can opt for potentially curative surgery, this therapeutic strategy is rarely feasible for those with distant metastasis or those undergoing relapse [5,6,7,8,9,10]. For these patients, a multifaceted, multidisciplinary approach is imperative, encompassing treatments like peptide receptor radionuclide therapy (PPRT), everolimus, somatostatin analogues, targeted therapies, and temozolomide-based chemotherapy regimens [5,6,7,8,9,10].

Temozolomide (TMZ) has carved a niche for itself as a potent alkylating chemotherapeutic agent, demonstrating activity against glioblastomas, melanomas, and neuroendocrine tumors [11]. Parallelly, bevacizumab, a humanized IgG1 monoclonal antibody designed against VEGF-A165, acts by indirectly inhibiting VEGFR-1 and VEGFR-2 upon blockade of VEGF-A165 [12]. This agent has showcased efficacy against an array of malignancies, including metastatic colorectal cancer, recurrent or metastatic lung cancer, ovarian cancer, glioblastoma, and cervical cancer, although its administration is occasionally marred by side effects such as delayed wound healing, bleeding, and intestinal perforation [13]. The phase 3 SWOG 0518 trial offers invaluable insights; this prospective study contrasted the therapeutic efficacy and safety of octreotide combined with bevacizumab versus octreotide combined with interferon in patients diagnosed with carcinoid tumors [14,15]. It was revealed that anti-VEGF antibodies could markedly decrease intratumoral VEGF concentrations, leading to significant tumor growth inhibition in murine models with transplanted endocrine tumors upon intraperitoneal administration [14]. Although the progression-free survival (PFS) exhibited by bevacizumab mirrored that of interferon, bevacizumab displayed a superior response rate, extended time to treatment failure (TTF), and a reduced incidence of side effects like fatigue [15]. Capturing the tumor cycle in low proliferating (slow-growing) neuroendocrine tumors is of paramount importance, and daily, low-dose continuous chemotherapy regimens might be aptly suited for this purpose [16,17,18]. Variations in dosing have been observed in studies focused on the metronomic application of TMZ [16,17,18]. This drug’s primary mechanism pivots around the inhibition of tumor angiogenesis by directly targeting endothelial cells in the tumor milieu and curtailing the mobilization of endothelial progenitor cells from the bone marrow [19]. Such a mechanism presents several advantages over traditional chemotherapy, particularly in delaying drug resistance onset and diminishing toxicity to the host [19]. Impressively, even at minimal oral concentrations of 20 mg/m^2^, TMZ manifests inhibitory actions [19]. Numerous studies have attested to its efficacy in treating neuroendocrine tumors, either as monotherapy or synergistically with other agents [17,18,19,20,21,22,23,24]. While a plethora of literature elucidates the efficacy of TMZ monotherapy or in tandem with capecitabine for metastatic neuroendocrine tumors [16,17,18,20,22,23,24], only a handful have delved into the combination of TMZ and bevacizumab [21,25]. There also exists a case report highlighting the dramatic radiological and clinical response observed in a patient with metastatic neuroendocrine carcinoma. This patient, after facing swift progression with the cisplatin–etoposide regimen, displayed a significant turnaround within five months upon transitioning to a combined regimen of TMZ and bevacizumab [26].

In the present study, our endeavor is to meticulously assess the efficacy and the spectrum of side effects associated with a combined regimen of bevacizumab and metronomic temozolomide (mTMZ) in patients diagnosed with metastatic neuroendocrine tumors.

## 2. Methods

### 2.1. Study Design

**Figure 1:**
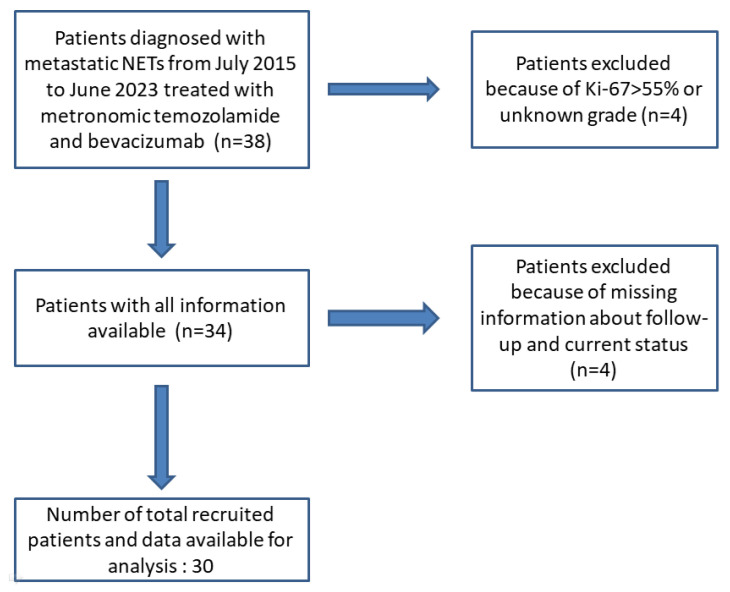
Flow diagram of patients identified and included in the final analysis.

This investigation is a retrospective analysis of prospectively captured data concerning 30 patients diagnosed with stage IV neuroendocrine tumors (either de novo or upon relapse). These patients were treated with mTMZ and bevacizumab and followed by Prof. Dr. Sezer Sağlam from July 2015 through to June 2023. Out of the 38 patients administered mTMZ and bevacizumab, only 30 were included for analysis in this study due to the reasons elucidated in Figure 1. We excluded patients exhibiting a Ki-67 value exceeding 55% [27,28]. Furthermore, to eliminate potential tumor heterogeneity, those with neuroendocrine carcinoma were not considered [27,28]. The cohort was stratified based on tumor grade (grade 1 and 2 vs. grade 3), Ki-67 levels (≤20%, 21–55%), and treatment outcomes with specific regard to PFS and OS.

Ethical clearance was procured from Istanbul Bilgi University (project code: 2021-40034-46).

### 2.2. Patients, Treatment, and Tissue Specimens

Enrollment criteria included patients aged 18 years or older diagnosed with NET, presenting with radiologically quantifiable metastatic disease as per the RECIST 1.1 guidelines. We included both paraffin-embedded and formalin-fixed tissue samples obtained from primary tumors or metastatic sites. Hospital-based specialist pathologists confirmed the NET diagnosis. A meticulous compilation of demographic details (including age, gender, prior treatments, and metastatic status), pathological attributes (encompassing the Ki-67 proliferative index and WHO grade), and imaging findings was undertaken. Regardless of the treatment sequence, patients having been administered the present treatment regimen either as the first line or subsequent lines were included. Prior therapies such as somatostatin analog, PPRT, 5-FU-based combinations, everolimus, or sunitinib did not preclude participation. Concomitant usage of somatostatin analogues was permissible.

Furthermore, individuals who had previously undergone local therapeutic interventions, including TARE, TACE, radiofrequency ablation, or SBRT, were encompassed. All participants were appraised for their performance based on the Eastern Cooperative Oncology Group Performance Status (ECOG PS), ranging between 0 to 2, and were expected to have a life expectancy extending beyond three months. Preliminary laboratory criteria were established to ensure optimal hematological, renal, and hepatic functionality. Exclusion criteria were rooted in the presence of systemic infections, coagulation abnormalities, or unmanaged chronic conditions.

Patients were routinely administered mTMZ (40 mg daily) alongside a combination regimen of bevacizumab 5 mg/kg every 21 days. The treatment persisted until the onset of intolerable side effects, disease advancement, or death. Comprehensive hematological assessments were instituted bi-weekly for the initial two months and subsequently tri-weekly (preceding bevacizumab administration) to monitor for significant adverse events.

### 2.3. Assessment of Tumor Response to Therapy and Survival Safety Profile

Tumor response to the current treatment was monitored every 12 weeks, and patients with regular three-month clinical follow-ups were included. Radiological response was evaluated by specialist radiologists at the cancer center using the Response Evaluation Criteria in Solid Tumors (RECIST) version 1.1. [29]. A “Complete Response” (CR) referred to the total absence of all tumor lesions. On the other hand, a “Partial Response” (PR) was characterized by a reduction of at least 30% in the aggregate of the greatest lesion diameters. If there was the emergence of new lesions or a growth of 20% or greater in the longest diameters from the beginning of treatment, it was categorized as “Progressive Disease” (PD). “Stable Disease” (SD) was indicated when there was a size increase of less than 20% or a reduction of less than 30% with no new lesion emergence. The “Objective Response Rate” (ORR) combined the rates of CR and PR. Meanwhile, the “Disease Control Rate” (DCR) encompassed CR, PR, and SD combined. 

We examined the duration of current treatment administration, the reasons for discontinuation of the treatment, and side effects. The follow-up period was defined as the time from disease diagnosis to the last follow-up or date of death. The duration from when mTMZ and bevacizumab treatment began to the point of disease progression or any cause of death was used to determine the progression-free survival (PFS). For patients who remained alive without disease progression, their data was censored as of their last known visit. The overall survival (OS) was characterized as the period between the commencement of current combined therapy and the date of death, regardless of the cause.

Adverse events were graded according to the National Cancer Institute Common Terminology Criteria for Adverse Events (NCI-CTCAE version 4) [30]. 

### 2.4. Statistical Analysis

Data were methodically processed using SPSS version 22.0. Continuous variables were represented using median values (interquartile range (IQR) or range), while categorical variables were presented as basic proportions. Kaplan–Meier techniques estimated the median PFS and OS. Subset analyses evaluated PFS and OS across parameters like Ki-67, tumor grade, and primary tumor location. Differences based on histological grade, Ki-67 indices, and primary tumor site were compared using the Kaplan–Meier methodology. A *p*-value below 0.05 was deemed indicative of statistical significance across all tests.

## 3. Results

### 3.1. Demographics, Tumor, and Treatment Characteristics

We evaluated 30 patients diagnosed with metastatic neuroendocrine tumors (NETs) who underwent treatment with mTMZ and bevacizumab from July 2015 to June 2023. The median follow-up duration was 45.8 months (11.7–98.8 months). Detailed clinicopathological characteristics of the patients are provided in Table 1.

### 3.2. Clinical Outcomes

**Figure 2:**
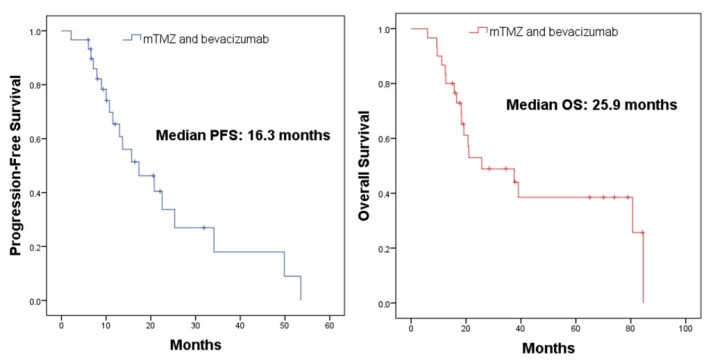
Survival analysis of patients treated with mTMZ and bevacizumab.

The median progression-free survival (PFS) was 16.3 months, and the median overall survival (OS) was 25.9 months (Figure 2). 

**Figure 3:**
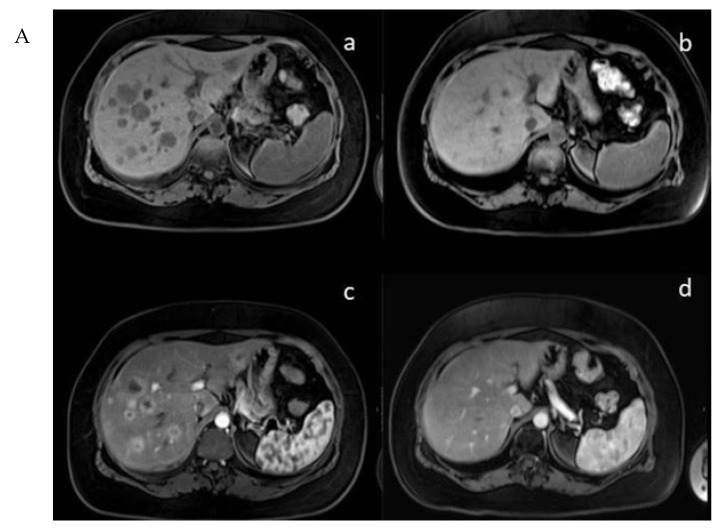

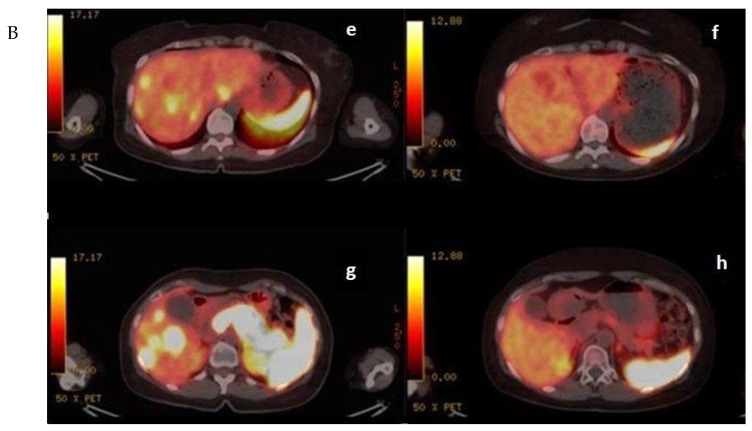
(**A**) (**upper**) Downstaging tumors (complete response) after metronomic TMZ–bevacizumab combination therapy. MRI images for the patient with metastatic NET before (**a**,**c**) and six months after treatment (**b**,**d**); (**B**) (**bottom**) downstaging tumors (complete response) after metronomic TMZ–bevacizumab combination therapy. Gallium-68 (Ga-68)-DOTATATE positron emission tomography (PET) images for the patient with metastatic NET before (**e,g**) and one year after treatment (**f**,**h**).

In the mTMZ + bevacizumab cohort of 30 patients, 4 (13.3%) exhibited a complete response (CR) (Figure 3), 15 (50.0%) demonstrated a partial response (PR), 7 (23.3%) had stable disease (SD), and 4 (13.3%) experienced progressive disease (PD). The overall objective response rate (ORR), combining CR and PR, reached 63.3% (19 patients), while the disease control rate (DCR), which includes CR, PR, and SD, stood at 86.7% (26 patients) (Table 2). In the course of our study evaluating the temozolomide–bevacizumab (mTMZ–bevacizumab) regimen for metastatic neuroendocrine tumors (NETs), treatment was continued until patients experienced intolerable side effects, disease progression, or death. Of the 30 patients included in our study, 18 patients reached mortality due to disease progression.

**Figure 4:**
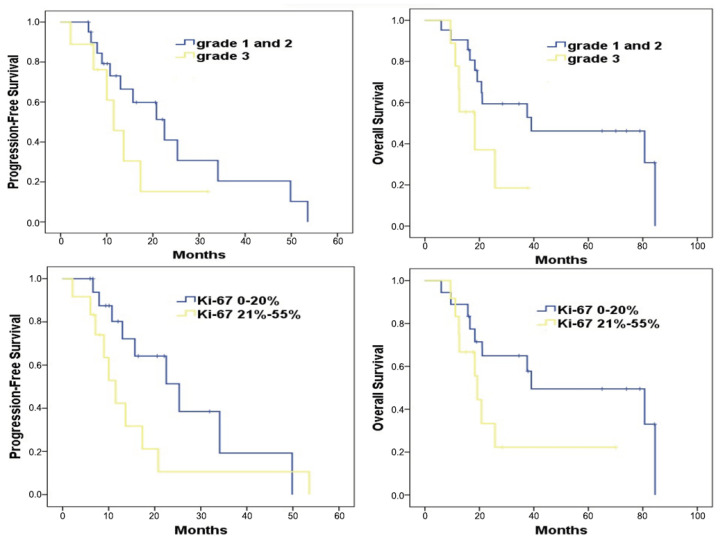
Survival analysis of patients treated with mTMZ and bevacizumab treatment according to the grade 1, 2 vs. 3 (**upper**) and Ki-67 groups (≤20%, 21–55%) (**bottom**).

In tumor grade stratification, patients with grade 1 and 2 tumors had a median PFS of 22.5 months, while those with grade 3 tumors experienced a median PFS of 11.4 months. This difference was not statistically significant (*p* = 0.17) (Figure 4; upper left). However, when examining overall survival, patients with grade 1 and 2 tumors had a significantly longer median OS of 39.0 months in contrast to the 18.2 months observed for grade 3 tumor patients (*p* = 0.04) (Figure 4; upper right).

With regard to Ki-67 levels, patients exhibiting a Ki-67 value of ≤20% showed a median PFS of 25.3 months (Figure 4; bottom left). Conversely, those with Ki-67 values ranging between 21% and 55% had a shorter median PFS of 11.4 months, a difference that was statistically significant (*p* = 0.03) (Figure 4; bottom right). When assessing overall survival in relation to Ki-67 values, the median OS durations stood at 39.9 months for those with a Ki-67 value of ≤20%, and 19.2 months for those with Ki-67 values between 21% and 55% (*p* = 0.07).

Due to the limited sample size of 30 patients, we were unable to stratify and analyze based on separate primary tumor locations, which precluded us from performing a detailed survival analysis.

### 3.3. Toxicity

Toxicity profiles of the patients are outlined in Table 3. The most commonly reported side effects, graded 1–2, were fatigue (26.4%), skin rash (20%), anemia, anorexia (both at 13.2%), followed by decreased lymphocyte count, nausea/vomiting, diarrhea, mucositis oral, pruritus, and hypertension (each reported in 10% of the patients). Grade 3 toxicities were relatively rare and were observed for decreased lymphocyte count, mucositis oral, pruritus, hypertension, and anal fistula. The overall rate of grade 3–4 toxicities was 20%, with all reported toxicities being grade 3. 

No grade 4 adverse events were documented. Some patients experienced specific effects necessitating treatment adjustments. Notably, 3 patients (10%) required a drug holiday. No dose reductions or drug discontinuation due to adverse effects were documented in our cohort. No treatment-related fatalities were reported.

## 4. Discussion

In this retrospective analysis of prospectively captured data concerning 30 patients with metastatic NETs (Ki-67 > 55%) treated with mTMZ and bevacizumab, we noted encouraging survival outcomes, effective disease control, and a favorable safety profile. These results indicate that while the mTMZ and bevacizumab regimen does come with a range of side effects, most are of low grade and manageable. The absence of grade 4 events and the relatively low incidence of grade 3 toxicities highlight the regimen’s favorable safety profile. The combination treatment demonstrated an overall response rate (ORR) of 63.3% and a disease control rate (DCR) of 86.7% (Table 2). Importantly, the median progression-free survival (PFS) and overall survival (OS) stood at 16.3 months and 25.9 months, respectively. Such findings are significant, especially when considering the diverse grades of tumors and varied Ki-67 levels represented in the cohort. It was discerned that patients with grade 1 and 2 tumors showed a comparatively longer PFS and OS than those with grade 3 tumors. Moreover, the Ki-67 index, a proliferation marker, played a discernible role in survival outcomes. Patients with a Ki-67 value of ≤20% experienced longer PFS and OS compared to those with Ki-67 values between 21% and 55%. The results also underscored the limitations due to the cohort size, which hindered a detailed survival analysis based on primary tumor locations. Based on the results from our study, it appears that this regimen could be considered as an alternative to the widely used temozolomide–capecitabine (CAPTEM) treatment for patients with metastatic NETs.

There are only two studies that align closely with our research in the literature [21,25]. Chan et al. administered temozolomide (TMZ) (150 mg/m^2^ per day on days 1 through 7 and days 15 through 21 repeated every 28 days) and bevacizumab (5 mg/kg per day on days 1 and 15 repeated every 28 days) to 34 patients diagnosed with metastatic NET. Of these, 10 patients (29.4%) were unable to continue with the treatment due to grade 3–4 side effects [21]. In another study, Koumarianou et al. administered mTMZ (100 mg/day), bevacizumab (7.5 mg/kg repeated every 21 days), and a somatostatin analog combined treatment to patients (Ki-67 < 20%). They reported that no patients discontinued treatment due to drug side effects [25]. However, compared to our study, Koumarianou’s study had a smaller patient sample (*n* = 15), a shorter follow-up period, and did not include any patients with a Ki-67 above 20% [25]. In our study, we did not observe any treatment discontinuation due to grade 3–4 side effects, nor did we detect any grade 4 side effects in patients (Table 3). 

As temozolomide–bevacizumab treatment is widely used in patients with GBM, many related studies are available in the literature [31,32,33,34]. One study administered TMZ (50 mg/m^2^) and bevacizumab (10 mg/kg) every 14 days to 32 patients with recurrent glioblastoma. Two patients (grade 4 hematotoxicity and pancreatitis) could not continue treatment due to side effects, and one patient died due to pneumonia [31]. Given the higher doses of TMZ treatment in this study compared to ours, this might explain the increase in the rate of grade 4 and 5 side effects observed. Another study involving geriatric patients with GBM compared mTMZ (40 mg/day) to standard TMZ treatment and observed no grade 4 side effects [32]. The reduced side effect profile of mTMZ treatment suggests it might be more tolerable than conventional treatments, potentially offering a better quality of life for patients. A phase I study dosed patients with recurrent GBM with mTMZ at varying doses and found a correlation between increasing dose and side effect severity [33]. Similarly, Chan et al. noted that grade 3–4 side effects were mostly linked to conventionally dosed TMZ treatment [21]. Another research study indicated that hypertension, a known side effect of bevacizumab, occurred in two patients but was manageable and did not warrant treatment cessation [25]. In our cohort of 30 patients treated with mTMZ and bevacizumab, the most common side effects were fatigue (26.4%), skin rash (20%), and anemia (13.2%). The overall grade 3–4 toxicity rate was 20%, which was solely attributed to grade 3 events. Treatment was interrupted for a week in two (6.7%) patients who developed grade 3 hyperbilirubinemia due to TMZ. The TMZ treatment was resumed after the bilirubin level normalized. One anal fistula developed in two (6.7%) patients due to bevacizumab treatment, leading to a one-month treatment interruption. In addition, two (6.7%) patients who developed grade 3 hypertension were managed with antihypertensive treatment, and treatment proceeded without interruption. 

In our study evaluating the efficacy of the temozolomide–bevacizumab (mTMZ–bevacizumab) regimen for metastatic neuroendocrine tumors (NETs), we observed a median progression-free survival (PFS) of 16.3 months and an overall survival (OS) of 25.9 months. This result is a significant improvement compared to the 11.0 months median PFS reported by Chan et al. [21], who studied 34 patients. Additionally, Chan et al. noted a PFS of 14.3 months in pancreatic NET patients and 7.3 months in carcinoid tumor patients, with a disease control rate (DCR) of 80.0%. Interestingly, Koumarianou et al. conducted a study on a combination treatment involving metronomic temozolomide, bevacizumab, and a long-acting octreotide in malignant neuroendocrine tumors [25]. They reported efficacy rates that contribute to our understanding of combination therapy in this field. While the specific PFS and OS metrics in Koumarianou et al.’s study are not explicitly stated, their findings on the efficacy of a similar combination regimen provide a relevant point of comparison for our results [25]. In contrast, Kunz et al.’s study reported a median PFS of 22.7 months for the CAPTEM regimen, with a notable overall survival of 58.7 months [20]. This highlights the efficacy of combination therapies in advanced pancreatic NETs. However, the design differences between our retrospective study and their randomized controlled trial (RCT) warrant a cautious approach in direct comparisons. The CAPTEM regimen’s effectiveness is further underscored by a meta-analysis by Arrivi et al., which involved 1818 patients and reported a DCR of 77%, with a wide-ranging median PFS of 4 to 38 months [23]. This variability in treatment outcomes reflects the heterogeneity of NETs. Furthermore, the OCLURANDOM trial conducted by Baudin et al. showed a PFS of 20.7 months with peptide receptor radionuclide therapy [34]. Although this PFS is higher than that observed in our study, it highlights the diverse therapeutic approaches and their varying efficacies in different NET subtypes. Akihiro et al. have also contributed to the understanding of NET treatment, particularly regarding the use of temozolomide-based therapies [35]. In their findings, temozolomide±capecitabine treatment is indicated as a chemotherapy regimen in patients with metastatic NETs, other than pancreatic NETs, providing further evidence of the evolving treatment landscape in this field. Our study offers a promising alternative to CAPTEM, especially for patient subgroups that might benefit more from an mTMZ–bevacizumab combination. The findings across various studies demonstrate the need for individualized treatment strategies based on tumor characteristics and patient profiles. Our research adds valuable insights into the treatment of metastatic NETs with mTMZ–bevacizumab. The need for more extensive research, particularly through larger RCTs, is evident to explore the full potential of this regimen. Such studies will enable a deeper understanding and optimization of treatment strategies for diverse patient populations within the NET spectrum.

Our study brings to light the potential of the temozolomide–bevacizumab (mTMZ–bevacizumab) regimen in treating metastatic neuroendocrine tumors (NETs) across different tumor grades and Ki-67 proliferation indices. Notably, patients with lower-grade tumors (grades 1 and 2) in our study exhibited prolonged progression-free survival (PFS), suggesting a favorable response to the treatment (grade 1-2 vs. grade 3: PFS: 22.5 vs. 11.4 months, respectively, *p* = 0.17; OS: 39.0 vs. 18.2 months, respectively, *p* = 0.04). This outcome aligns with the established understanding that lower-grade NETs typically have a more indolent course and may respond better to treatment. In contrast, those with grade 3 tumors, known for their more aggressive behavior, experienced more rapid disease progression, highlighting the challenges in treating high-grade NETs. In terms of Ki-67 proliferation index, a key marker of tumor aggressiveness, patients in our study with a Ki-67 index of ≤20% showed promising PFS and overall survival (OS) (Ki-67 ≤20% vs. Ki-67 21%-55%: PFS: 25.3 vs. 11.4 months, respectively, *p* = 0.03; OS: 39.9 vs. 19.2 months, respectively, *p* = 0.07). This observation is significant as it suggests that our regimen is particularly effective against tumors with lower proliferative rates. The efficacy of the regimen in less proliferative tumors (lower Ki-67 index) underscores the importance of considering tumor biology in treatment planning. However, when we compare these findings with the existing literature, including studies by Chan et al. [21] and Koumarianou et al. [25], we encounter certain limitations. These studies, while informative, often lack detailed subgroup analyses based on tumor grades and Ki-67 indices, making direct comparisons with our results challenging. Chan et al., for instance, reported on the effectiveness of a different therapeutic approach but did not provide a detailed breakdown by tumor grade or Ki-67 index, which is crucial for a nuanced understanding of treatment outcomes. Moreover, studies on the CAPTEM regimen, another recognized treatment for neuroendocrine tumors, present with a high degree of patient heterogeneity [16,17,18,20,22,23,24]. This variability in patient profiles, encompassing a range of tumor grades and Ki-67 indices, adds complexity to any direct comparison with our results. For instance, the CAPTEM studies have shown efficacy in a broad spectrum of NETs but often include a mix of tumor grades and proliferative rates, which can obscure specific insights relevant to particular patient subgroups. Therefore, while our study contributes valuable insights, particularly regarding the efficacy of the mTMZ–bevacizumab regimen in lower-grade and less proliferative NETs, there is a clear need for further exploration in more homogenous and larger cohorts. Future studies should aim to stratify patients more distinctly based on tumor grades and Ki-67 indices to discern the most effective therapeutic strategies for each subgroup. Such targeted research would be instrumental in refining treatment approaches and improving outcomes for patients with different subtypes of metastatic NETs.

Temozolomide is a drug whose effectiveness is known and cannot be used continuously due to toxicity. Various studies in the literature have examined temozolomide monotherapy as well as combination therapies that include temozolomide [18,21,36,37]. Although temozolomide demonstrates high efficacy in NET patients, standard-dose usage has been associated with a treatment discontinuation rate of up to 55% due to grade 3–4 side effects [21,24,25,36,37]. Thus, new treatment regimens are needed for temozolomide–bevacizumab based low toxicity therapies. These new approaches should aim to mitigate high-grade side effects in the treatment of NETs, ensure the continuity of drug therapy, and enhance PFS and DCR. In our analysis, while the efficacy of metronomic temozolomide (mTMZ) was a focal point, the significant role of bevacizumab in our treatment regimen warrants a detailed discussion. Bevacizumab, as an anti-angiogenic agent targeting VEGF-A, plays a critical role in hindering tumor growth by inhibiting the formation of new blood vessels. This mechanism is especially pertinent in neuroendocrine tumors, which are typically highly vascularized. The study by Yao et al. in a Phase III trial, comparing depot octreotide with interferon alfa-2b against octreotide with bevacizumab, underscores the effectiveness of bevacizumab in managing carcinoid tumors, highlighting its potential in combination therapies like ours [15]. However, the use of bevacizumab in cancer treatment is nuanced. While it has shown promise in certain types of cancer, its effectiveness varies across different malignancies. For instance, Capozzi et al. (2016) discussed the role of antiangiogenic therapy in pancreatic neuroendocrine tumors, indicating the potential benefits and limitations of agents like bevacizumab [38]. These studies highlight the need for a careful, personalized approach when incorporating bevacizumab into cancer treatment regimens. Furthermore, the impact of bevacizumab on inflammation is complex. Some studies suggest that it can induce inflammation, adding a layer of complexity to its use in cancer therapy [39]. Conversely, other research indicates its potential in reducing inflammation, which could be beneficial in controlling tumor progression [40]. This dichotomy necessitates a deeper understanding of bevacizumab’s multifaceted effects and careful patient monitoring during treatment. Our study’s findings, in the context of the broader literature, suggest that the mTMZ–bevacizumab combination is a promising therapeutic option for metastatic NETs. However, the role of bevacizumab, with its varying impact on angiogenesis and inflammation, as well as its differential efficacy across cancer types, should be carefully considered. This underscores the importance of ongoing research to optimize the use of bevacizumab in combination therapies, ensuring the best possible outcomes for patients with neuroendocrine tumors.

Taken together, these data highlight the therapeutic potential of both mTMZ–bevacizumab and CAPTEM regimens in managing neuroendocrine tumors. The choice between these regimens should be individualized based on patient profiles, disease characteristics, and potential toxicities. As therapeutic strategies evolve, ongoing research and comparative studies are vital for refining treatment protocols.

### Limitations

Nature of the study: our analysis, while retrospective, is based on a prospective cohort, which offers a structured and systematic collection of data over time. This methodology does offer advantages over purely retrospective designs in terms of data consistency and quality. However, it still brings forth potential biases related to patient selection and interpretation. While the efficacy we report for mTMZ and bevacizumab aligns with other retrospective trials, randomized controlled trials would be more definitive in establishing efficacy and safety.

Comparison to other trials: although our study demonstrated efficacy similar to other trials using conventional schedules of TMZ and bevacizumab, it is essential to note that direct comparisons might not always be accurate due to variations in study design, patient populations, and other confounding factors.

Sample size: the limited number of patients in our study can affect the statistical power and generalizability of our findings. A larger cohort would provide more robust data and potentially highlight subtler effects or rare adverse events.

Tumor localization heterogeneity: in evaluating the efficacy of the temozolomide–bevacizumab (mTMZ–bevacizumab) regimen in metastatic neuroendocrine tumors (NETs), our study encountered a limitation due to the sample size, which restricted our ability to stratify and analyze the data based on primary tumor locations. The significance of tumor localization in NETs is well documented, as different sites of origin can exhibit distinct biological behaviors and responses to treatment. The lack of stratification by tumor location in our research means that we might have overlooked variations in treatment response that are specific to the primary site of the tumor. This is a critical consideration in NET treatment, where the efficacy of a regimen can vary significantly depending on whether the primary tumor is in the pancreas, gastrointestinal tract, lungs, or another location. Each site can present unique challenges and response patterns to therapeutic interventions. Consequently, while our findings provide valuable insights into the overall effectiveness of the mTMZ–bevacizumab regimen, they should be interpreted with an understanding of this constraint. The general applicability of our results across all NET subtypes may be limited, and the nuances associated with different tumor localizations could not be fully explored in our study. Future research with larger cohorts that allow for detailed stratification based on tumor location is essential. Such studies would enable a more comprehensive and precise understanding of how different NET subtypes respond to various treatment regimens, including mTMZ–bevacizumab. This approach is crucial for advancing personalized medicine in NET treatment, ensuring that therapeutic strategies are tailored to the unique characteristics of tumors based on their site of origin.

Duration of follow-up: The follow-up period’s length and consistency can influence the accurate assessment of long-term outcomes. 

## 5. Conclusions

In our investigation of mTMZ and bevacizumab for the treatment of neuroendocrine tumors (NETs), the regimen demonstrated promising efficacy markers comparable to other established treatments. Notably, the progression-free survival (PFS) and disease control rate (DCR) observed in this study are encouraging. Such a safety profile suggests that this regimen could be particularly beneficial for older, fragile patients who may find conventional dosages challenging. While the outcomes, particularly in relation to the tumor grade and Ki-67 index, provide valuable insights into patient stratification, it is crucial to interpret these findings in the context of the study’s limitations. The retrospective analysis of our prospective cohort, though methodologically robust, indicates a need for further randomized controlled trials to solidify our understanding. As the search for optimized treatment strategies for NETs continues, the results presented here contribute to the growing body of evidence underscoring the potential role of the current study. With future studies, the precise positioning of this regimen in the therapeutic landscape for NETs can be further refined.

## Figures and Tables

**Table 1:** Patients and tumor characteristics of patients treated with tem/bev (*n* = 30).

		mTMZ and Bevacizumab n (%)
**Age median (min–max)**		50 (38–77)
**Gender**	Female Male	14 (46.7%) 16 (53.3%)
**Age groups**	<50 50–70 >70	15 (50.0%) 9 (30.0%) 6 (20.0%)
**PS (ECOG)**	0 1 2	18 (60.0%) 8 (26.7%) 4 (13.3%)
Number of comorbidities *	0 1 2	18 (56.7%) 11 (36.7%) 2 (6.7%)
**Site (primary)**	Pancreas GIS Lung Unknown	12 (40.0%) 7 (23.3%) 10 (33.3%) 1 (3.4%)
**Tumor grade**	Grade 1 Grade 2 Grade 3	3 (10.0%) 18 (60.0%) 9 (30.0%)
**Prior surgery (curative)**	Yes No	6 (20.0%) 24 (80.0%)
**Metastatic sites**	Liver Extra-liver Liver and Extra-liver	9 (30.0%) 8 (26.7%) 13 (43.3%)
**Ki-67 (%)**	≤20 21–55	18 (60.0%) 12 (40.0%)
**Ki-67 (%)**	Median (min-max)	15 (2–50)
**Functioning**	Yes No	3 (10.0%) 27 (90.0%)
**Previous treatment at metastatic stage, n (%)**	Surgery (metastasectomy) Liver-Directed Therapy Somatostatin analogues PRRT Cytotoxic chemotherapy Everolimus	3 (10.0%) 4 (13.3%) 10 (33.3%) 11 (36.7%) 8 (26.7%) 1 (3.3%)
**Previous lines of tem/bev**	0 1 ≥2	11 (36.7%) 14 (46.7%) 5 (16.7%)
**Concurrent octreotide use**	Yes No	14 (46.7%) 16 (53.3%)

* hypertension, diabetes, coronary heart disease, chronic kidney disease, cerebrovascular disease, chronic obstructive pulmonary disease; tem/bev: metronomic temozoloamide and bevacizumab.

**Table 2:** Best responses according to the current treatment.

Response Category, n (%)	mTMZ + Bevacizumab (*n* = 30)
Complete response (CR)	4 (13.3%)
Partial response (PR)	15 (50.0%)
Stable disease (SD)	7 (23.3%)
Progressive disease (PD)	4 (13.3%)
Objective response rate (CR + PR)	19 (63.3%)
Disease control rate (CR + PR + SD)	26 (86.7%)

**Table 3:** Adverse effects of mTMZ and bevacizumab regimen.

Toxicity Type	mTMZ/Bevacizumab (*n* = 30)		
	Grade—n (%)		
	1, 2	3	4
Anemia	4 (13.2%)	-	-
Platelet count decreased	2 (6.6%)	-	
Neutrophil count decreased	2 (6.6%)	-	
Lymphocyte count decreased	3 (12%)	1 (3.3%)	-
Fatigue	8 (26.4%)	-	-
Nausea/vomiting	3 (10%)	-	-
Constipation	1 (3.3%)	-	-
Headache	2 (6.6%)	-	-
Diarrhea	3 (10%)	-	-
Anorexia	4 (13.2%)	-	-
Abdominal pain	2 (6.6%)	-	-
Epistaxis	2 (6.6%)	-	
Fever	1 (3.3%)	-	-
Mucositis oral	3 (10%)	1 (3.3%)	-
Pruritis	3 (10%)	2 (6.6%)	-
Proteinuria	2 (6.6%)	-	-
Hypertension	3 (10%)	1 (3.3%)	-
Dyspnea	1 (3.3%)	-	-
Skin rash	5 (20%)	-	-
Anal fistula	1 (3.3%)	2 (6.6%)	-
Blood bilirubin increased	2 (6.6%)	2 (6.6%)	-
Increased ALP	2 (6.6%)	-	-
Increased AST	3 (10%)	-	-
Increased ALT	2 (6.6%)	-	-
Hemorrhage	1 (3.3%)	-	-
Thrombosis or embolism	-	-	-
Dehydration	3 (10%)	-	-
Grade 3–4 toxicity rate	20% (only grade 3)		
Dose reduction	N/A		
Drug holiday	3 (10%)		
Drug discontinuation	N/A		

## Data Availability

The datasets generated during and/or analyzed during the current study are available from the corresponding author upon reasonable request.
